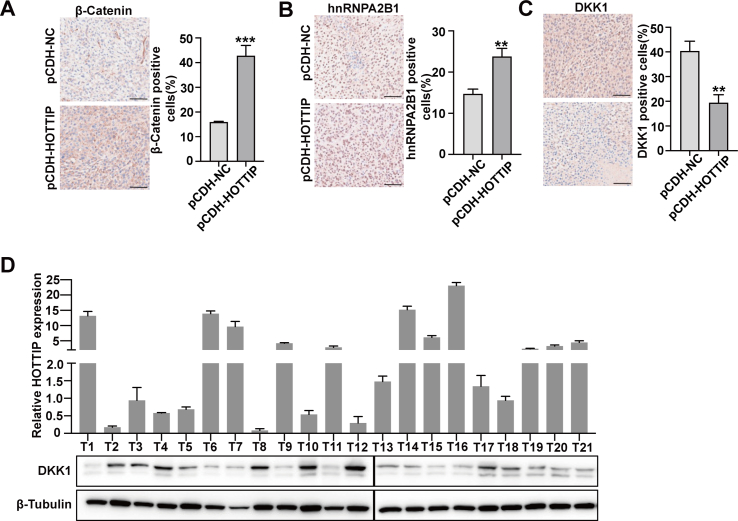# LncRNA HOTTIP facilitated tumor growth *via* stimulating the hnRNPA2B1/DKK1/Wnt/β-catenin regulatory axis in hepatocellular carcinoma

**DOI:** 10.1016/j.gendis.2023.05.012

**Published:** 2023-07-04

**Authors:** Weiqiang Zeng, Chuanjian Shi, Liqiang Deng, Weiming Fu, Jinfang Zhang

**Affiliations:** a Cancer Center, Shenzhen Hospital (Futian) of Guangzhou University of Chinese Medicine, Shenzhen, Guangdong 518000, China; b Guangdong Provincial Key Laboratory of New Drug Screening, School of Pharmaceutical Sciences, Southern Medical University, Guangzhou, Guangdong 510515, China

Hepatocellular carcinoma (HCC) has become the fifth leading cause of cancer mortality worldwide in the past decade.^1^ Dysregulated long noncoding RNAs (lncRNAs) are closely associated with its occurrence and progression. Recently identified lncRNA named “HOXA transcript at the distal tip” (HOTTIP) has been considered as an oncogene in multiple cancers. However, the mechanism underlying HOTTIP-mediated hepatocarcinogenesis remains mostly unknown.

Using the TCGA database, HOTTIP was observed to be increased in HCC tissues (Fig. S1A). The qRT-PCR examination also showed that it was significantly up-regulated in 30 paired HCC specimens (Fig. 1A), which was consistent with the result from the database. Then we monitored the expression profile of HOTTIP in a panel of HCC cell lines, and the results showed HOTTIP was significantly up-regulated in most HCC cell lines, except the HepG2 and Hep3B cells (Fig. S1B). The stable HOTTIP overexpressing HepG2 and Hep3B cells, and the HOTTIP silencing PLC/PRF/5 cells were therefore developed (Fig. S1C–E). Based on the examination of CCK-8 assays and colony formation, HOTTIP overexpression accelerated HCC cell growth, and HOTTIP knockdown impaired cell proliferation (Fig. 1B, C; Fig. S1F, G). Moreover, the nude xenograft mice model demonstrated HOTTIP overexpression dramatically stimulated tumor growth *in vivo* (Fig. 1D; Fig. S1H–J). These data indicate that HOTTIP acts as an oncogenic regulator to facilitate tumor growth *in vitro* and *in vivo.*

**Figure 1:**
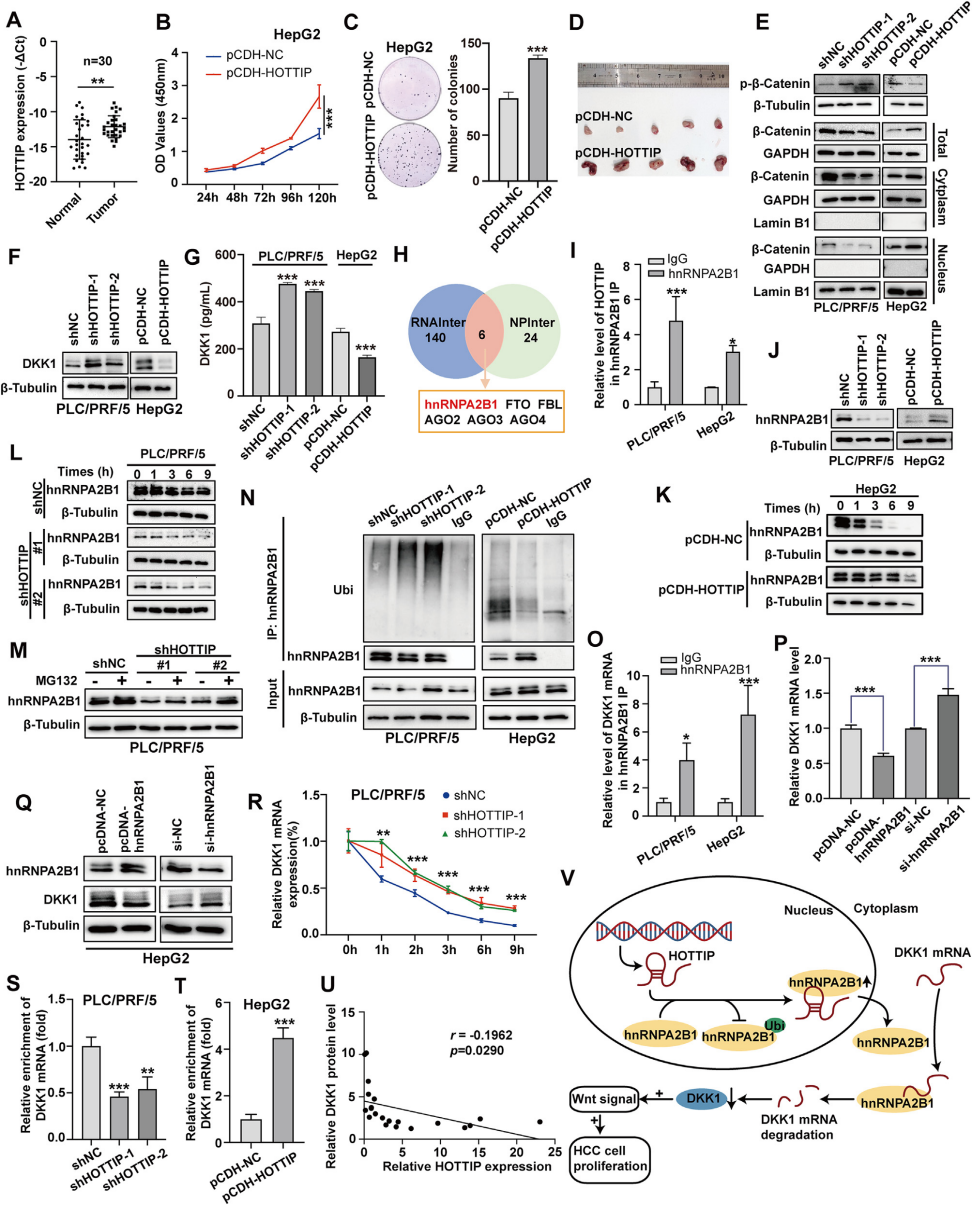
HOTTIP recruits hnRNPA2B1 by post-transcriptional modification and negatively regulates DKK1 to activate the Wnt/β-catenin signaling. **(A)** Analysis of HOTTIP expression by qRT-PCR in 30 pairs of HCC tissues. The expression levels were normalized to GAPDH. **(B)** The effects of HOTTIP overexpression on HCC cell growth were determined by CCK8 assays. **(C)** The effects of HOTTIP overexpression on cell proliferation were evaluated by colony formation assays. **(D)** The nude mice which were subcutaneously injected with HOTTIP overexpressing and corresponding control cells were sacrificed and the tumor tissues were collected. **(E)** The protein levels of phosphorylation of β-catenin at Ser33, Ser37, and Thr41, total and subcellular β-catenin were analyzed after knockdown or overexpression of HOTTIP. **(F)** The protein level of DKK1 was analyzed after knockdown or overexpression of HOTTIP by Western blot. **(G)** The DKK1 level in cell supernatant was assessed after knockdown or overexpression of HOTTIP by ELISA. **(H)** The Venn diagram showing the potential HOTTIP-binding proteins between RNAInter 4.0 and NPInter 5.0 bioinformatics software. **(I)** The interaction between HOTTIP and hnRNPA2B1 was validated by RIP assay. **(J)** The protein level of hnRNPA2B1 was determined after the knockdown or overexpression of HOTTIP in HCC cells. **(K, L)** With 75 μg/mL CHX treatment, the protein level of hnRNPA2B1 was measured after knockdown or overexpression of HOTTIP in HCC cells at indicated time points. **(M)** The protein level of hnRNPA2B1 was detected by Western blot in HOTTIP-silencing PLC/PRF/5 cells after treatment with 25 μM MG132. **(N)** Western blot followed by immunoprecipitation to detect the ubiquitylation of hnRNPA2B1 in HOTTIP-silencing PLC/PRF/5 and HOTTIP-up-regulated HepG2. **(O)** The interaction between hnRNPA2B1 and DKK1 mRNA was validated by RIP assay. **(P, Q)** The protein and mRNA levels of DKK1 were detected after the knockdown or overexpression of hnRNPA2B1 in HCC cells. **(R)** With 1 μg/mL actinomycin D treatment, the mRNA level of DKK1 was measured after the knockdown of HOTTIP in HCC cells at indicated time points. **(S, T)** The enrichments of DKK1 mRNA were examined in the hnRNPA2B1 recruited complex with knockdown or overexpression of HOTTIP. **(U)** Correlation analysis showed a negative relationship between HOTTIP (x) and DKK1 protein levels (y) in 21 HCC tissues. The data were shown as means ± SD (*n* = 3). ^∗^*P* < 0.05, ^∗∗^*P* < 0.01, ^∗∗∗^*P* < 0.001, versus their representative controls. **(V)** Schematic overview of HOTTIP/hnRNPA2B1/DKK1/Wnt/β-catenin regulatory axis in HCC.

It has been reported that HOTTIP could form an R-loop structure at TAD boundaries of the Wnt/β-catenin gene to promote β-catenin transcriptional activation in leukemia.^2^ We wondered whether the Wnt/β-catenin signal participated in the HOTTIP-mediated HCC tumor growth. The Wnt/β-catenin signaling luciferase reporter Topflash was transfected into HCC cells, and it was found that the silence of HOTTIP reduced the luciferase activity, while overexpression of HOTTIP exhibited an inverted trend (Fig. S2A, B). HOTTIP knockdown repressed the total and subcellular β-catenin expression and increased phosphorylation of β-catenin at Ser33, Ser37, and Thr41, while opposite results were achieved by HOTTIP overexpression (Fig. 1E). The similar phenomenon was observed in the transcription of several β-catenin target genes (Fig. S2C, D). The antagonists might be therapeutic strategies for HCC patients with activated Wnt signaling. The twelve common Wnt pathway-related inhibitors were selected and their expression was examined (Table S2). Of these, five antagonists were up-regulated by HOTTIP knockdown (Fig. S2E). These candidates were further validated in the HOTTIP overexpressing HCC cells, and Dickkopf-1 (DKK1) was identified as the most inhibited one (Fig. S2F). As a secreted protein inhibiting Wnt/β-catenin signaling, DKK1 expression was promoted by HOTTIP knockdown while it was suppressed by HOTTIP overexpression in cellular protein and culture medium (Fig. 1F, G). The further rescue study indicated that DKK1 overexpression significantly attenuated the HOTTIP-facilitated tumor cell growth and Wnt/β-catenin signaling activation (Fig. S2G–J). These results indicate that DKK1 participates in the HOTTIP-mediated cell proliferation and Wnt/β-catenin signaling.

Considering that lncRNAs may interact with transcription factors and hence modulate gene transcription, we applied two online bioinformatical programs,^3^^,^^4^ NPInter v5.0 and RNAinter v4.0 (Table S3), to predict the targets of HOTTIP (Fig. 1H). Among the potential candidates, heterogeneous nuclear ribonucleoprotein A2B1 (hnRNPA2B1) was verified to physically interact with HOTTIP by RNA immunoprecipitation (RIP) examination (Fig. 1I). We next evaluated its expression and the results showed that it remained consistent whether HOTTIP knockdown or overexpression at the transcriptional level (Fig. S3A). Surprisingly, the protein expression of hnRNPA2B1 was suppressed by HOTTIP silence while it was promoted by HOTTIP overexpression at the translational level (Fig. 1J). We, therefore, suspected that HOTTIP mediated the hnRNPA2B1 expression in a post-transcriptional manner. To validate this hypothesis, the protein synthesis inhibitor cycloheximide (CHX) was introduced and the half-life of hnRNPA2B1 was monitored. We observed the stability of hnRNPA2B1 was impaired by HOTTIP knockdown while it was scarcely affected by HOTTIP overexpression (Fig. 1K, L; Fig. S3B, C). The ubiquitin-proteasome system is considered the central player in protein stability. The inhibitor of proteasome MG132 was found to alleviate the suppressive effect of HOTTIP knockdown on hnRNPA2B1 (Fig. 1M). We further utilized hnRNPA2B1 antibody to pull down endogenous hnRNPA2B1 proteins and examined their ubiquitin modification. The results revealed an increased ubiquitin modification in HOTTIP knockdown cells and a decreased ubiquitination in HOTTIP overexpression cells (Fig. 1N). All these data suggest that HOTTIP recruits hnRNPA2B1 and prevents hnRNPA2B1 ubiquitination and degradation, which leads to promoting its expression.

The hnRNPA2B1 has been reported to shuttle between the nucleus and cytoplasm and subsequently regulate RNA stability.^5^ We, therefore, monitored hnRNPA2B1 expression in subcellular fraction with HOTTIP knockdown or overexpression. Although the nuclear hnRNPA2B1 expression remained consistent, the cytoplasmic expression was increased by HOTTIP overexpression while it was decreased by HOTTIP knockdown (Fig. S3D, E). Our RIP examination showed that DKK1 mRNA was preferentially enriched in the hnRNPA2B1-recruited complex (Fig. 1O), suggesting the interaction between DKK1 mRNA and hnRNPA2B1. DKK1 expression was suppressed by hnRNPA2B1 overexpression while it was promoted by hnRNPA2B1 knockdown at the mRNA (Fig. 1P) and protein levels (Fig. 1Q). Moreover, the stability of DKK1 was monitored and its protein stability was scarcely influenced by HOTTIP (Fig. S3F, G). Regarding mRNA stability, HOTTIP knockdown stabilized the DKK1 mRNA, whereas HOTTIP overexpression destroyed the mRNA stability of DKK1 (Fig. 1R; Fig. S3H). We further verified the interplay between hnRNPA2B1 and DKK1 mRNA closely depended on HOTTIP regulation by RIP-qPCR examination (Fig. 1S, T). Furthermore, we monitored the HOTTIP/hnRNPA2B1/DKK1 regulatory axis in tumor specimens derived from nude mice. By immunohistochemistry staining, the increased expression of β-catenin and hnRNPA2B1 while the decreased expression of DKK1 was observed in the HOTTIP overexpression group (Fig. S4A–C). Finally, we detected HOTTIP and DKK1 protein levels in fresh HCC clinical tissues, and the results showed HOTTIP expression is negatively correlated with DKK1 protein expression (Fig. 1U; Fig. S4D). According to these results, HOTTIP negatively regulates DKK1 expression via physically interacting with hnRNPA2B1.

Collectively, the mechanistic model was proposed to illustrate the underlying molecular mechanism of HOTTIP-mediated tumor growth (Fig. 1V). Of which, HOTTIP was found to directly bind to hnRNPA2B1 and maintain its stability via disrupting the ubiquitin-dependant degradation. The hnRNPA2B1 was validated to interact with DKK1 mRNA and repressed its expression, which led to activation of the Wnt/β-catenin pathway and tumor growth. The findings provide a novel insight into the mechanism of HOTTIP and may develop a potential molecular-targeted therapy for HCC with activated Wnt/β-catenin signaling.

## Ethics declaration

The animal experiments and treatments were approved by the Institutional Animal Care and Use Committee (IACUC) of Southern Medical University (SMU), Guangzhou, China (Approval No. L2018187). Informed consent was obtained where applicable. The clinical tissues were obtained with informed consent and the relative research was approved by the Joint Chinese University of Hong Kong-New Territories Ease Cluster Clinical Research Ethics Committee.

## Author contributions

WMF and JFZ contributed to the study design and supervision. WQZ, CJS, and LQD performed experiments and data analysis. WQZ and CJS wrote the manuscript. All authors read and approved the final manuscript.

## Conflict of interests

The authors declare that they have no competing interests.

## Figures and Tables

**figs1:**
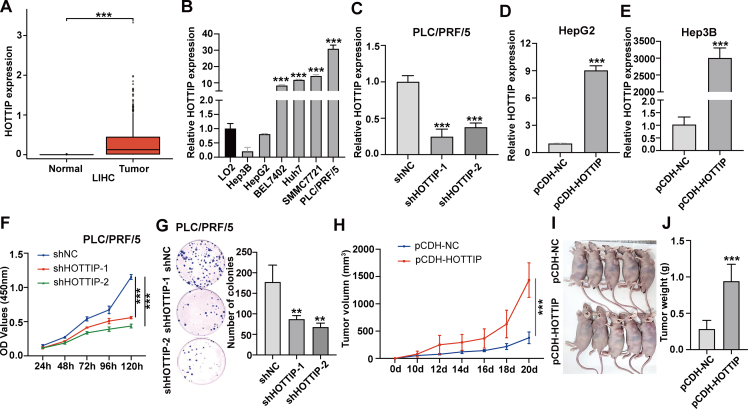


**figs2:**
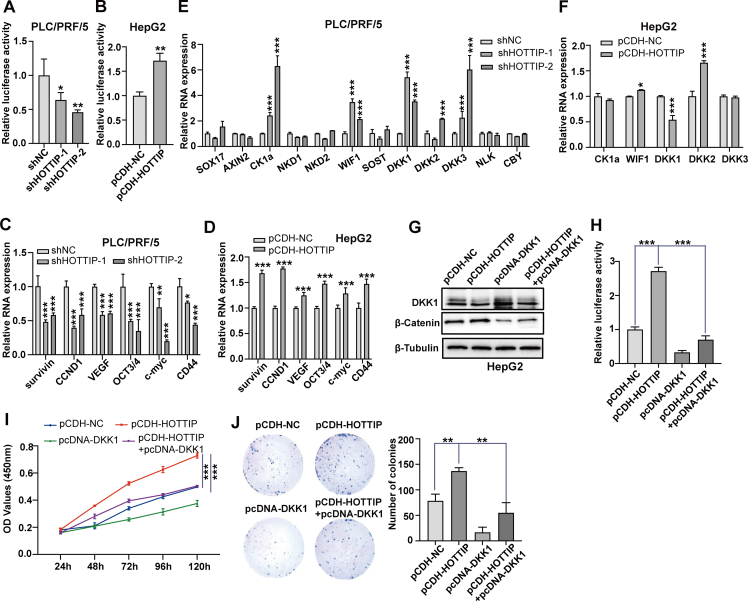


**figs3:**
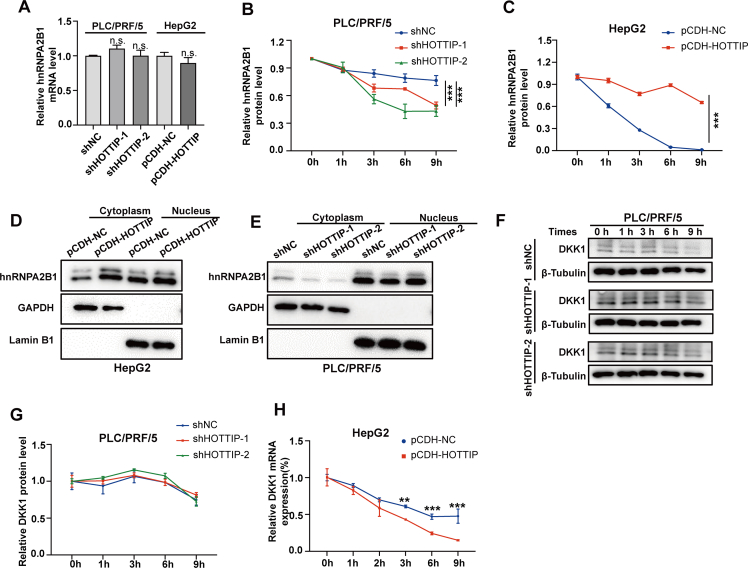


**figs4:**