# Supplementary material for: LncRNA HOTTIP facilitated tumor growth via stimulating the hnRNPA2B1/DKK1/Wnt/β-catenin regulatory axis in hepatocellular carcinoma
Source: Genes Dis. 2023 Jul 4;11(4):101013. doi: 10.1016/j.gendis.2023.05.012 (PMC10933460; doi:10.1016/j.gendis.2023.05.012)
Supplement: Multimedia component 1 [file mmc1.docx]

**Supplementary information for**

**LncRNA HOTTIP facilitated tumor growth *via* stimulating the hnRNPA2B1/ DKK1/ Wnt/β-catenin regulatory axis in hepatocellular carcinoma**

Wei-Qiang Zeng,^2#^ Chuan-Jian Shi,^2#^ Li-Qiang Deng,^2^ Wei-Ming Fu,^2^ Jin-Fang Zhang^1^

**Corresponding Author:** Dr. Jin-Fang Zhang, Shenzhen Hospital (Futian) of Guangzhou University of Chinese Medicine, email: [zhangjf06@gzucm.edu.cn](mailto:zhangjf06@gzucm.edu.cn).

**Contents:**

**Materials and methods** Page 2

**Table S1.** Oligonucleotide sequences in this study Page 9

**Table S2.** Negative regulators in Wnt/β-catenin signal Page 11

**Fig. S1** HOTTIP accelerated tumor growth *in vitro* and *in vivo*. Page 14

**Fig. S2** HOTTIP suppressed the DKK1 expression to activate Wnt/β-catenin signaling and promote tumor cell growth. Page 15

**Fig. S3** HnRNPA2B1 actively participated in the trans-regulation between HOTTIP and DKK1. Page 16

**Fig. S4** The correlation of HOTTIP with DKK1 expression *in vivo* tissues. Page 17

**Materials and methods**

**Reagents and antibodies**

MG-132 (T2154) was obtained from TargetMol (MA, USA). Cycloheximide (CHX, HY-12320) was obtained from MedChemExpress (MCE, NJ, USA). Actinomycin D (Act-D, D1515) was purchased from Sigma-Aldrich (MO, USA). The primary antibodies used in this study were as follows: anti-β-catenin antibody (Cat.NO.:8480S, Cell Signaling Technology, MA USA), anti-phospho-β-catenin antibody (Cat.NO.:9561S, Cell Signaling Technology, MA USA), anti-GAPDH antibody (Cat.NO.:5174S, Cell Signaling Technology), anti-Ubiquitin antibody (Cat.NO.:3933S, Cell Signaling Technology), anti-hnRNPA2B1 antibody (Cat.NO.:ab31645, Abcam, MA USA), anti-DKK1 antibody (Cat.NO.:ab109416, Abcam), anti-Lamin B1 antibody (Cat.NO.:T40003F, Abmart, shanghai, China) and anti-β-Tubulin antibody (Cat.NO.:M20023, Abmart). The secondary anti-rabbit antibodies (Cat. No.: AP132P) and anti-mice antibodies (Cat. No.: AP127P) were purchased from Merck Millipore (MIT, USA).

**Cell culture**

Human HCC cell lines including HepG2, PLC/PRF/5, Hep3B, BEL7402, Huh7, SMMC7721, normal liver cell LO2, and the human embryonic kidney cell line HEK293T were purchased from American Type Culture Collection. All the cell lines were cultured in Dulbecco’s Modified Eagle medium containing 10% fetal bovine serum, 100 U/ml penicillin, and 100 μg/ml streptomycin at 37℃ in a 5% CO_2_ atmosphere.

**Database analysis**

The HOTTIP expression data were extracted from the TCGA-LIHC database (<https://cancergenome.nih.gov/>) and analyzed by R software (3.5.3). The HOTTIP-targeted proteins were predicted by RNAInter 4.0 ([http://rnainter.org](http://rnainter.org/)) and NPInter 5.0 (<http://bigdata.ibp.ac.cn/npinter5/>).

**Transfection and stable cell lines generation**

To develop the DKK1 or hnRNPA2B1 overexpressing cell lines, full-length DKK1 ([NM_012242.4](https://www.ncbi.nlm.nih.gov/nuccore/NM_012242.4)) or hnRNPA2B1 (NM_002137.4) was inserted into pcDNA3.1 vector to generate the overexpression plasmids. These plasmids were transfected into HCC cells using Lipofectamine 3000. The small interfering RNA (siRNA) for hnRNPA2B1 and negative control were transfected into HCC cells by using Lipofectamine 3000 reagent. For generating HOTTIP overexpressing and silencing cell lines, full-length HOTTIP or empty lentiviral vector were inserted into pCDH-CMV-MCS-EF1-Puro-GFP, and two pairs of siRNAs for HOTTIP were cloned into the lentiviral vector pGLV3/H1/GFP-Puro and a scrambled control was employed as the negative control (shNC). Using the retrovirus system, the plasmids including pCDH-NC, pCDH-HOTTIP, shNC and shHOTTIP-1/2 were introduced with psPAX2 and pMD2G into HEK293T cells, and the supernatant was collected at 48 h. With filtration, these virus particles infected Hep3B, HepG2, and PLC/PRF/5, and the stable cell lines were developed with puromycin (4 μg/ml) screening. All the sequences of primers and siRNAs were shown in Table S1.

**Cell viability assays**

Cells were seeded into 96-well plates at the density of 800 cells per well, and the Cell Counting Assay Kit-8 (CCK-8, Cat. NO.: C0005, TargetMol, MA, USA) solution was added. With incubation for another 1h, the absorbance was determined by a Hybrid Multi-Mode Microplate Reader (Tecan, Switzerland) at 450 nm.

**Colony formation assay**

Cells were seeded into 6-well plates at a density of 200-1000 cells per well and maintained for two weeks. The colonies were fixed with methanol and stained with Giemsa staining solution (Beyotime, Shanghai, China). The colony numbers were recorded by the ImmunoSpot analyzer (CTL, USA).

**RNA isolation, reverse transcription and quantitative real-time PCR (qRT-PCR) examination**

Total RNA was extracted from cells using Total RNA Isolation Kit (Cat. NO.: RE-03014, FOREGENE, Chengdu, China), and complementary DNA was transcribed with PrimeScript RT Reagent Kit (Cat. NO. RR036A, Takara, Japan). All qRT-PCR examination was performed using Power up SYBR Green Master Mix (Cat. NO.: A25742, Thermo Fisher Scientific, Waltham, MA, USA) in the ABI QuantStudio6 system (Thermo Fisher, USA). The relative expression levels of genes normalizing to GAPDH were determined using the 2^–ΔΔCt^ method. The primer sequences were shown in Table S1.

**Western blot**

Total protein was isolated using Radio Immunoprecipitation Assay (RIPA buffer, Cat. NO.: P0013B, Beyotime) including phosphatase inhibitor (P003, New Cell & Molecular Biotech) and protease (P1045, Beyotime). The cytoplasmic and nuclear protein were obtained using Nuclear and Cytoplasmic Protein Extraction Kit (Cat. NO.: P0028, Beyotime). All the protein samples were quantified with BCA Assay Kit (Cat. NO.: 023227, Thermo Fisher Scientific) and separated *via* 10% SDS-PAGE. After transferring protein to the PVDF membrane (Cat. NO.: SLGV004SL-1, Millipore, MA, USA), the membranes were blocked in 5% defatted milk for 1h at room temperature and then probed with the primary antibodies overnight at 4℃. Subsequently, the PVDF membranes were incubated with the relative HRP-conjugated secondary antibody (Merck Millipore) at a 1:5000 dilution for 1 h at room temperature. Finally, the images were captured by chemiluminescent imaging system (FluorChem R, USA).

**Enzyme-linked immunosorbent assays (ELISA)**

The concentration of DKK1 protein in the condition medium was assessed by Human DKK1 ELISA (Cat. NO.: EK0867, Boster, Shanghai, China) according to the manufacturer’s instruction. The absorbance was determined by a Hybrid Multi-Mode Microplate Reader (Tecan, Switzerland) at 450 nm.

**Immunohistochemistry (IHC)**

The specimens were fixed in 4% paraformaldehyde, dehydrated, and embedded in paraffin. Then the sections were incubated with the primary antibodies as follows: anti-β-catenin antibody (1:100; Cell Signaling Technology), anti-hnRNPA2B1 antibody (Cat.NO.: ab31645, Abcam), and anti-DKK1 antibody (Cat.NO.: ab109416, Abcam) at 4℃ for overnight and then counterstained with hematoxylin (Cat. NO.: BA-4041, BaSo, Zhuhai, China). The images were captured with 40× magnification and visualized analysis was performed by Image J.

**Luciferase activity assays**

The cells were seeded in 24-well plates and transfected with luciferase reporter TOPflash and pRL-TK plasmids simultaneously. The Dual-Luciferase® Reporter Assay System (E1910, Promega, Madison, WI, USA) was used to evaluate relative luciferase activity 48 h after lysing the cells according to the manufacturer’s instruction in a Hybrid Multi-Mode Microplate Reader. The renilla luciferase activity derived from pRL-TK plasmid was used as a loading control for normalization.

**Clinical samples analysis**

The primary HCC specimens and non-tumor tissues were collected from the Prince of Wales Hospital (Hong Kong). All tissue samples were identified by pathologists. All patients provided written informed consents and relative studies were approved by Joint

Chinese University of Hong Kong-New Territories Ease Cluster Clinical Research Ethics Committee.

**Animal study**

The operations and usage of animals were approved by the Institutional Animal Care and Use Committee (IACUC) of Southern medical university (Guangzhou, China, Approval No. L2019140). The nude BALB/c mice were subcutaneously injected with 1×10^6^ stable Hep3B cells with HOTTIP overexpression or control cells (*n* = 5 per group). The tumor volumes (V) were measured every two days and calculated as: *V*=0.5×*S*^2^×*L* (*S*, the shortest millimeters; *L*, the longest millimeters). Three weeks later, the xenograft tumors were finally dissected from mice for further immunohistochemistry investigation.

**RNA immunoprecipitation (RIP)**

Cells were collected and treated with NP-40 lysis buffer (P0013F, Beyotime) supplemented with protease inhibitor (P1005, Beyotime), RNase inhibitor (R0102, Beyotime) and 1mM DTT at 4°C for 15 min. The 10% of the supernatant was used as input, and others were incubated with A/G magnetic beads and anti-hnRNPA2B1 antibodies or IgG overnight. The co-precipitated RNAs were incubated with Proteinase K (ST533, Beyotime) for 1 h at 55°C. Total RNAs were extracted by TRIzol reagent (T9109, Takara) to detect HOTTIP and DKK1 mRNA enrichment by qRT-PCR.

**Co-immunoprecipitation (Co-IP)**

MG132 was applied to cells at a concentration of 20 μM for 6 hours and then these cells were lysed in a cold IP lysis wash buffer containing protease inhibitors and RNase inhibitors. The supernatants were incubated with A/G magnetic beads binding to anti-hnRNPA2B1 antibodies or IgG overnight at 4°C. After being washed with cold lysis buffer for three times, the proteins were eluted for further western blotting analysis.

**Protein stability assay**

75 μg/ml cycloheximide (CHX, HY-12320) was added to HCC cells for the indicated periods, and the DKK1 and hnRNPA2B1 protein levels were determined by Western blotting.

**RNA stability assay**

Cells were treated with 1 μg/ml Actinomycin D (Act-D, D1515) and incubated at the indicated times to evaluate RNA stability. The DKK1 expression was examined by qRT-PCR after extracting total RNA.

**Statistical analysis**

The results were presented as mean ± SD from at least three times separate experiments. The Student’s t-test for two groups or One-way ANOVA for multiple comparisons were performed in the data statistical analysis using Graphpad prism software version 8.0. Data were considered statistically significant when P-value was less than 0.05. **p* < 0.05, ***p* < 0.01, ****p* < 0.001.

**Table S1.** Oligonucleotide sequences in this study

| **Gene** | **Forward primer (5’-3’)** | **Reverse primer (5’-3’)** |
| --- | --- | --- |
| GAPDH | GCACCACCAACTGCTTAGCA | TCTTCTGGGTGGCAGTGATG |
| DKK1 | CCTTGAACTCGGTTCTCAATTCC | CAATGGTCTGGTACTTATTCCCG |
| HnRNPA2B1 | ATTGATGGGAGAGTAGTTGAGCC | AATTCCGCCAACAAACAGCTT |
| HOTTIP | CCTAAAGCCACGCTTCTTTG | TGCAGGCTGGAGATCCTACT |
| Survivin | CCACCGCATCTCTACATTCAAG | CAAGTCTGGCTCGTTCTCAGTG |
| Oct3/4 | TCGAGAACCGAGTGAGAGGC | CACACTCGGACCACATCCTTC |
| CCND1 | CTGGAGGTCTGCGAGGAACA | CCTTCATCTTAGAGGCCACGAA |
| VEGF | AGGGCAGAATCATCACGAAGT | AGGGTCTCGATTGGATGGCA |
| CD44 | CTGCCGCTTTGCAGGTGTA | CATTGTGGGCAAGGTGCTATT |
| c-myc | GGCTCCTGGCAAAAGGTCA | CTGCGTAGTTGTGCTGATGT |
| SOX17 | GTGGACCGCACGGAATTTG | GGAGATTCACACCGGAGTCA |
| Axin2 | CAACACCAGGCGGAACGAA | GCCCAATAAGGAGTGTAAGGACT |
| Ck1a | AGTGGCAGTGAAGCTAGAATCT | CGCCCAATACCCATTAGGAAGTT |
| NKD1 | GGGAAACTTCACTCCAAGCC | CTCCCGATCCACTCCTCGAT |
| NKD2 | GAGGACCAGTGTCCCCTACAG | CTCCGTCATCTGCGCTGAG |
| WIF1 | TCTCCAAACACCTCAAAATGCT | GACACTCGCAGATGCGTCT |
| SOST | ACACAGCCTTCCGTGTAGTG | GGTTCATGGTCTTGTTGTTCTCC |
| DKK2 | CTCACAGATCGGCAGTTCG | ATGCCAGTCCTTGGTACATGC |
| DKK3 | AGGACACGCAGCACAAATTG | CCAGTCTGGTTGTTGGTTATCTT |
| NLK | CGCAAAAATGATGGCGGCTTA | CCCAGGGTTTAACATGGCTG |
| CBY | TCTTTGGGAATACGTTCAGTCCG | CCAGGTTCATAGTCGGGGA |
| **The shRNA and siRNA sequences used for plasmid construction** | | |
| shHOTTIP-1 Forward | GATCCGGCACTTTATATGCTGTAATTCAAGAGATTACAGCATATAAAGTGCCTTTTTT | |
| shHOTTIP-1 Reverse | AATTAAAAAAGGCACTTTATATGCTGTAATCTCTTGAATTACAGCATATAAAGTGCCG | |
| shHOTTIP-2 Forward | GATCCGTACGGAAGTTCCATTAATTTCAAGAGAATTAATGGAACTTCCGTACTTTTTT | |
| shHOTTIP-2 Reverse | AATTAAAAAAGTACGGAAGTTCCATTAATTCTCTTGAAATTAATGGAACTTCCGTACG | |
| si-hnRNPA2B1 | GAAAUACCAUACCAUCAAUTT | |
|  | AUUGAUGGUAUGGUAUUUCTT | |

**Table S2.** Negative regulators in Wnt/β-catenin signal

| **Genes** | **Cancer types** | **References** |
| --- | --- | --- |
| SOX17 | Hepatocellular carcinomaBreast cancer | ^1, 2^ |
| AXIN2 | Medulloblastomas  Colorectal cancer | ^3, 4^ |
| CK1a | Colorectal cancer | ^5^ |
| NKD1 | Colorectal cancer Hepatoblastomas | ^6, 7^ |
| NKD2 | Osteoasrcoma Hepatocellular carcinoma | ^8, 9^ |
| WIF1 | Bladder cancer Breast cancer | ^10, 11^ |
| SOST | Retinoblastoma | ^12^ |
| DKK1 | Renal cell carcinoma  Colorectal cancer | ^13, 14^ |
| DKK2 | Renal cell carcinoma | ^15^ |
| DKK3 | Lung adenocarcinoma  Renal cell carcinoma | ^16, 17^ |
| NLK | Hepatocellular carcinoma Colorectal cancer | ^18, 19^ |
| CBY | NasopharyngealGastric cancer | ^20, 21^ |

1. Fu, DY, Wang, ZM, Li, C, Wang, BL, Shen, ZZ, Huang, W*, et al.* (2010). Sox17, the canonical Wnt antagonist, is epigenetically inactivated by promoter methylation in human breast cancer. *Breast Cancer Res Treat* **119**: 601-612.

2. Jia, Y, Yang, Y, Liu, S, Herman, JG, Lu, F, and Guo, M (2010). SOX17 antagonizes WNT/β-catenin signaling pathway in hepatocellular carcinoma. *Epigenetics* **5**: 743-749.

3. Koch, A, Hrychyk, A, Hartmann, W, Waha, A, Mikeska, T, Waha, A*, et al.* (2007). Mutations of the Wnt antagonist AXIN2 (Conductin) result in TCF-dependent transcription in medulloblastomas. *Int J Cancer* **121**: 284-291.

4. Wu, ZQ, Brabletz, T, Fearon, E, Willis, AL, Hu, CY, Li, XY*, et al.* (2012). Canonical Wnt suppressor, Axin2, promotes colon carcinoma oncogenic activity. *Proc Natl Acad Sci U S A* **109**: 11312-11317.

5. Li, B, Orton, D, Neitzel, LR, Astudillo, L, Shen, C, Long, J*, et al.* (2017). Differential abundance of CK1α provides selectivity for pharmacological CK1α activators to target WNT-dependent tumors. *Sci Signal* **10**.

6. Guo, J, Cagatay, T, Zhou, G, Chan, CC, Blythe, S, Suyama, K*, et al.* (2009). Mutations in the human naked cuticle homolog NKD1 found in colorectal cancer alter Wnt/Dvl/beta-catenin signaling. *PLoS One* **4**: e7982.

7. Koch, A, Waha, A, Hartmann, W, Hrychyk, A, Schüller, U, Waha, A*, et al.* (2005). Elevated expression of Wnt antagonists is a common event in hepatoblastomas. *Clin Cancer Res* **11**: 4295-4304.

8. Zhao, S, Kurenbekova, L, Gao, Y, Roos, A, Creighton, CJ, Rao, P*, et al.* (2015). NKD2, a negative regulator of Wnt signaling, suppresses tumor growth and metastasis in osteosarcoma. *Oncogene* **34**: 5069-5079.

9. Wang, D, Zhang, S, Chen, Y, Hu, B, and Lu, C (2018). Low expression of NKD2 is associated with enhanced cell proliferation and poor prognosis in human hepatocellular carcinoma. *Hum Pathol* **72**: 80-90.

10. Tang, Y, Simoneau, AR, Liao, WX, Yi, G, Hope, C, Liu, F*, et al.* (2009). WIF1, a Wnt pathway inhibitor, regulates SKP2 and c-myc expression leading to G1 arrest and growth inhibition of human invasive urinary bladder cancer cells. *Mol Cancer Ther* **8**: 458-468.

11. Veeck, J, Wild, PJ, Fuchs, T, Schüffler, PJ, Hartmann, A, Knüchel, R*, et al.* (2009). Prognostic relevance of Wnt-inhibitory factor-1 (WIF1) and Dickkopf-3 (DKK3) promoter methylation in human breast cancer. *BMC Cancer* **9**: 217.

12. Wu, T, Wang, LN, Tang, DR, and Sun, FY (2017). SOST silencing promotes proliferation and invasion and reduces apoptosis of retinoblastoma cells by activating Wnt/β-catenin signaling pathway. *Gene Ther* **24**: 399-407.

13. Hirata, H, Hinoda, Y, Nakajima, K, Kawamoto, K, Kikuno, N, Ueno, K*, et al.* (2011). Wnt antagonist DKK1 acts as a tumor suppressor gene that induces apoptosis and inhibits proliferation in human renal cell carcinoma. *Int J Cancer* **128**: 1793-1803.

14. Qi, L, Sun, B, Liu, Z, Li, H, Gao, J, and Leng, X (2012). Dickkopf-1 inhibits epithelial-mesenchymal transition of colon cancer cells and contributes to colon cancer suppression. *Cancer Sci* **103**: 828-835.

15. Hirata, H, Hinoda, Y, Nakajima, K, Kawamoto, K, Kikuno, N, Kawakami, K*, et al.* (2009). Wnt antagonist gene DKK2 is epigenetically silenced and inhibits renal cancer progression through apoptotic and cell cycle pathways. *Clin Cancer Res* **15**: 5678-5687.

16. Wang, Z, Ma, LJ, Kang, Y, Li, X, and Zhang, XJ (2015). Dickkopf-3 (Dkk3) induces apoptosis in cisplatin-resistant lung adenocarcinoma cells via the Wnt/β-catenin pathway. *Oncol Rep* **33**: 1097-1106.

17. Ueno, K, Hirata, H, Majid, S, Chen, Y, Zaman, MS, Tabatabai, ZL*, et al.* (2011). Wnt antagonist DICKKOPF-3 (Dkk-3) induces apoptosis in human renal cell carcinoma. *Mol Carcinog* **50**: 449-457.

18. Jung, KH, Kim, JK, Noh, JH, Eun, JW, Bae, HJ, Xie, HJ*, et al.* (2010). Targeted disruption of Nemo-like kinase inhibits tumor cell growth by simultaneous suppression of cyclin D1 and CDK2 in human hepatocellular carcinoma. *J Cell Biochem* **110**: 687-696.

19. Yasuda, J, Tsuchiya, A, Yamada, T, Sakamoto, M, Sekiya, T, and Hirohashi, S (2003). Nemo-like kinase induces apoptosis in DLD-1 human colon cancer cells. *Biochem Biophys Res Commun* **308**: 227-233.

20. Cai, CF, Ye, GD, Shen, DY, Zhang, W, Chen, ML, Chen, XX*, et al.* (2018). Chibby suppresses aerobic glycolysis and proliferation of nasopharyngeal carcinoma via the Wnt/β-catenin-Lin28/let7-PDK1 cascade. *J Exp Clin Cancer Res* **37**: 104.

21. Li, FQ, Chiriboga, L, Black, MA, Takemaru, KI, and Raffaniello, RD (2019). Chibby is a weak regulator of β-catenin activity in gastric epithelium. *J Cell Physiol* **234**: 1871-1879.

**Supplementary Figure**


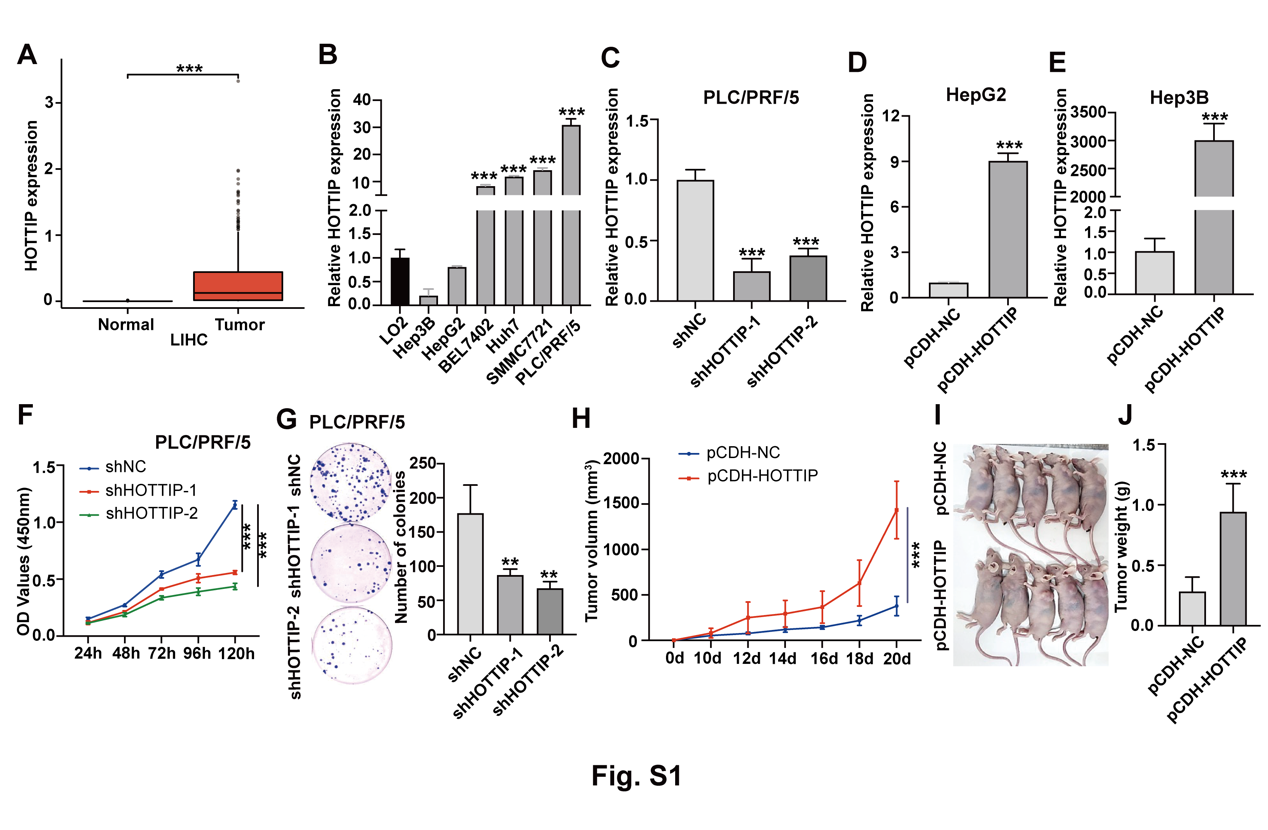


**Fig. S1 HOTTIP accelerated tumor growth *in vitro* and *in vivo*.** (A) Data on HOTTIP expression was obtained from the TCGA-LIHC dataset in the TCGA database, including 50 normal liver tissues and 374 HCC tissues; (B) The RNA level of HOTTIP was examined in a panel of HCC cell lines; (C) HOTTIP was significantly suppressed in the silencing PLC/PRF/5 cells; (D-E) HOTTIP was obviously upregulated in the overexpressing HepG2 and Hep3B cells; (F-G) The effects of HOTTIP knockdown on cell growth were determined by CCK8 assays and colony formation assays; (H-J) The tumor volume and weight data were evaluated from nude mice. The data were shown as means ± SD (*n*=3). *, *p*<0.05; **, *p*<0.01; ***, *p*<0.001; *vs* their representative controls.

**
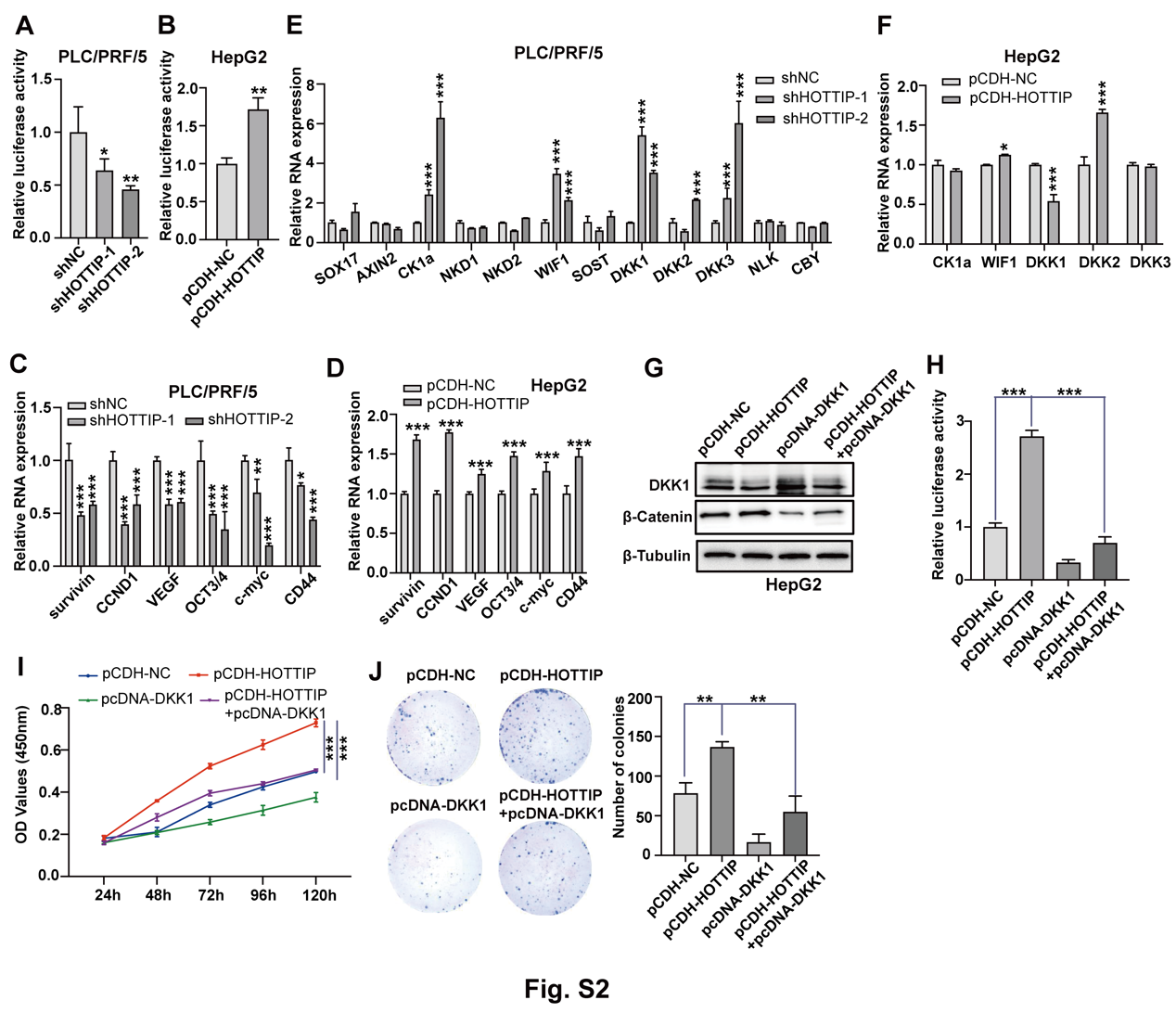
**

**Fig. S2 HOTTIP suppressed the DKK1 expression to activate Wnt/β-catenin signaling and promote tumor cell growth.** (A-B) The luciferase activities of TOPflash were measured after knockdown or overexpression of HOTTIP; (C-D) The expression of β-catenin target gene was monitored after knockdown or overexpression of HOTTIP; (E) The RNA levels of Wnt pathway antagonists were examined in HOTTIP silencing HCC cells; (F) The RNA levels of Wnt pathway antagonists were further examined in HOTTIP overexpressing HCC cells; (G) With the ectopic expression of DKK1, the protein levels of DKK1 and β-catenin were determined in the HOTTIP-overexpressing cells; (H) The luciferase activities of TOPflash were measured after ectopic expression of DKK1 and HOTTIP in the HCC cells; (I) The cell viability was detected by CCK8 assay after overexpression of DKK1 and HOTTIP in the HCC cells; (J) The colony formation was detected after overexpression of DKK1 and HOTTIP in the HCC cells. The data were shown as means ± SD (*n*=3). *, *p*<0.05; **, *p*<0.01; ***, *p*<0.001; *vs* their representative controls.


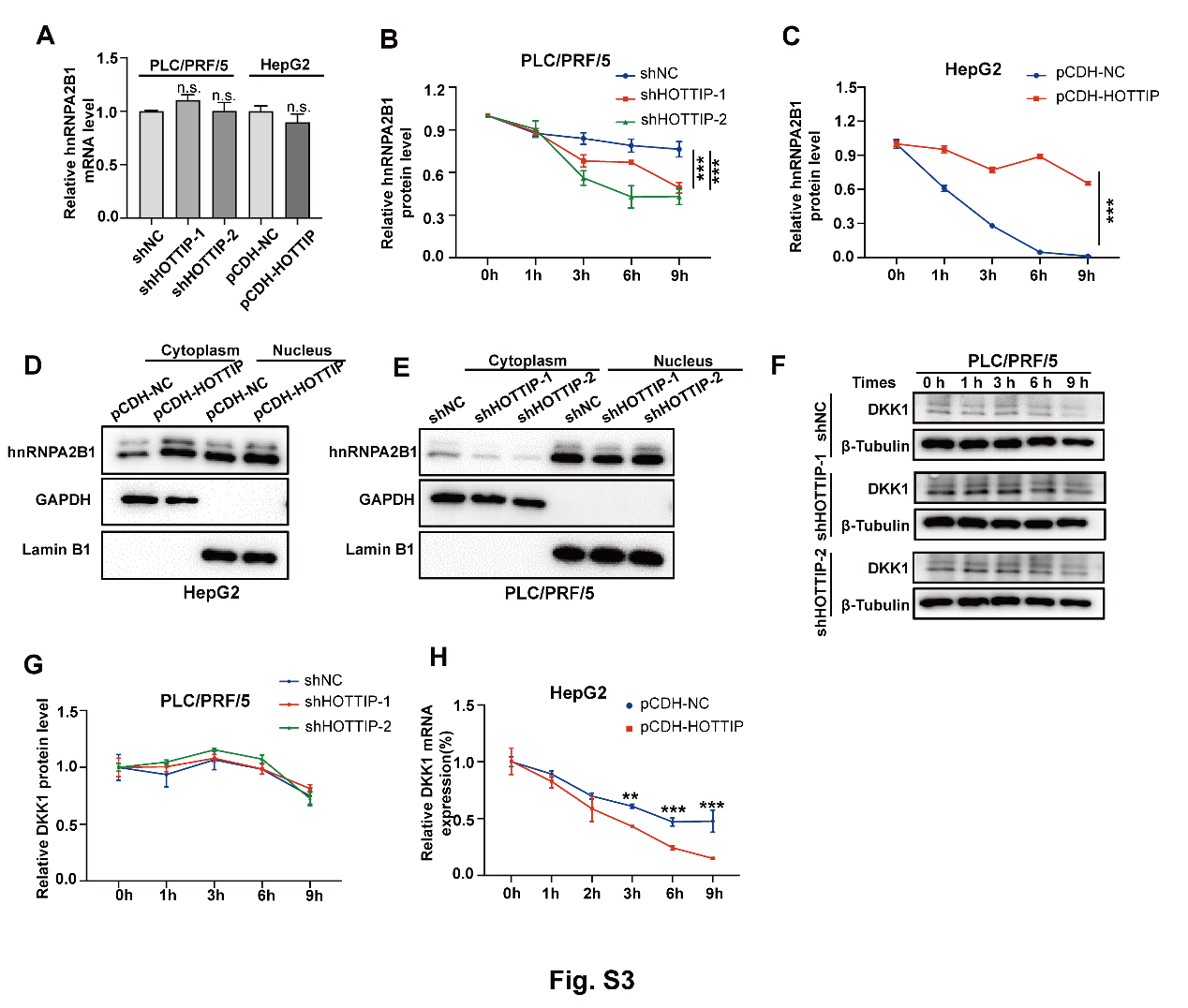


**Fig. S3 HnRNPA2B1 actively participated in the trans-regulation between HOTTIP and DKK1.** (A) The RNA level of hnRNPA2B1 was examined after knockdown or overexpression of HOTTIP in HCC cells; (B-C) The quantitative expression of hnRNPA2B1 was analyzed in the HOTTIP silencing or overexpressing HCC cells with 75 μg/ml CHX treatment; (D-E) The protein levels of cytoplasmic and nuclear hnRNPA2B1 were analyzed after knockdown or overexpression of HOTTIP; (F-G) With 75 μg/ml CHX treatment, the protein level of DKK1 was examined after knockdown of HOTTIP in HCC cells at indicated time points and the quantitative analyses; (H)With 1 μg/ml Actinomycin D treatment, the mRNA level of DKK1 was measured after overexpressing HOTTIP in HCC cells at indicated time points. The data were shown as means ± SD (*n*=3). *n.s.* no significance; **, *p*<0.01; ***, *p*<0.001; *vs* their representative controls.


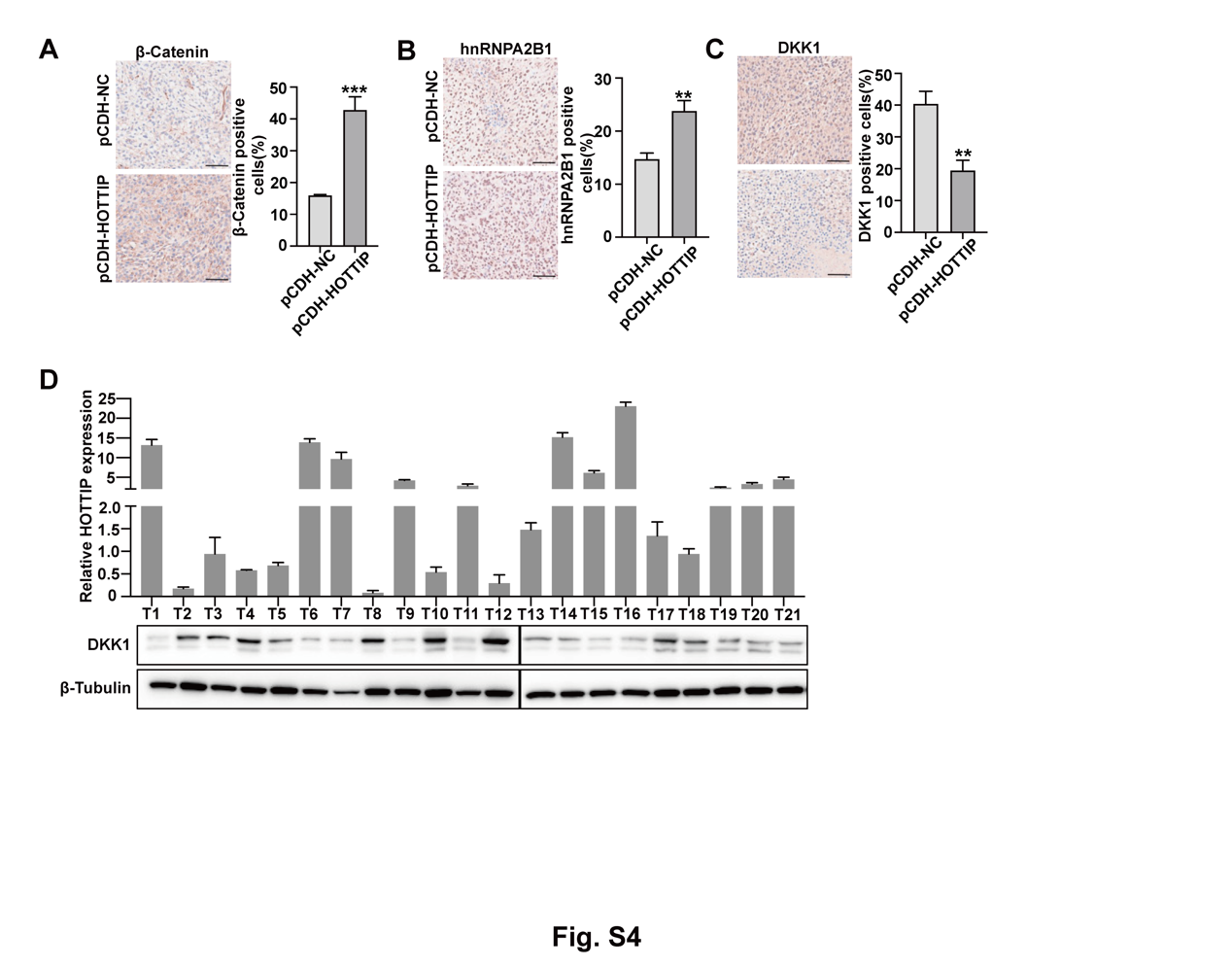


**Fig. S4 The correlation of HOTTIP with DKK1 expression *in vivo* tissues.** (A-C) The expression of β-catenin, hnRNPA2B1 and DKK1 were examined in the xenograft tumors sections by immunohistochemistry staining; (D) The HOTTIP RNA level and DKK1 protein were examined in 21 HCC tissues. GAPDH was used as the loading control for HOTTIP expression and β-Tubulin was used as the loading control for DKK1 protein level; Scale bar=25μm. The data were shown as means ± SD (*n*=3). **, *p*<0.01; ***, *p*<0.001; *vs* their representative controls.
